# Game-changing insights on vertebral skeletal stem cells in bone metastasis and therapeutic horizons

**DOI:** 10.32604/or.2023.046174

**Published:** 2023-11-15

**Authors:** QIUQIANG CHEN, XIAOLEI ZHAO, WENXUE MA

**Affiliations:** 1 Key Laboratory for Translational Medicine, The First Affiliated Hospital, Huzhou University School of Medicine, Huzhou, 313000, China; 2 Department of Urology, Huaihe Hospital of Henan University, Kaifeng, 475004, China; 3 Department of Medicine, Moores Cancer Center, Sanford Stem Cell Institute, University of California San Diego, La Jolla, 92093, USA

**Keywords:** Vertebral skeletal stem cells (vSSCs), Stem cell research, Metastasis, Breast, prostate, and lung cancers, Spinal metastasis, Matthew Greenblatt, Genetic expressions

## Abstract

Greenblatt and his team have unveiled vertebral skeletal stem cells (vSSCs) as a critical player in the landscape of bone metastasis. This commentary delves into the transformative discoveries surrounding vSSCs, emphasizing their distinct role in bone metastasis compared to other stem cell lineages. We illuminate the unique properties and functions of vSSCs, which may account for the elevated susceptibility of vertebral bones to metastatic invasion. Furthermore, we explore the exciting therapeutic horizons opened by this newfound understanding. These include potential interventions targeting vSSCs, modulation of associated signaling pathways, and broader implications for the treatment and management of bone metastasis. By shedding light on these game-changing insights, we hope to pave the way for novel strategies that could revolutionize the prognosis and treatment landscape for cancer patients with metastatic bone disease.

## Introduction

Stem cell research, a field rife with potential, has always held a magnetic allure for the scientific community. Especially within the context of bone health and disease, the promises stemming from these cells can potentially revolutionize our understanding and provide hope for numerous patients battling debilitating conditions. However, despite our progress, there remain puzzling gaps in our comprehension, especially concerning the specific mechanisms and players that govern bone physiology and pathology. One such enigmatic player is the vertebral skeletal stem cell (vSSC), which has lately drawn significant attention.

Recent research spearheaded by Sun et al. at Weill Cornell Medicine has illuminated that these vSSCs, responsible for the formation of our spine’s vertebral bones, are distinct from other bone-producing stem cells [1]. Even more compelling is their discovery that these cells secrete a protein, milk fat globule epidermal growth factor 8 (MFGE8), which appears to amplify the propensity of tumor cells to metastasize to the spine [2]. This revelation challenges not only our current understanding of cancer cell metastasis but also illuminates new therapeutic directions in both orthopedics and oncology.

The conventional perspective attributes the prevalence of spinal metastases to the specialized structure of the spinal bone marrow, which includes vascular sinusoids [3] and a relatively abundant blood flow within the vertebral bodies [4]. However, this long-standing paradigm left many questions unanswered and failed to explain all observed phenomena. Liu et al. identified genetic changes, such as differentially expressed genes (DEGs), within the metastatic microenvironment. These changes may promote metastasis, enhance survival, and facilitate the growth of cancer cells in the spine, regardless of the cancer type [5]. Kaplan et al.’s concept aligns with a prior study that underscores the significance of interactions between cancer cells and the bone marrow surrounding potential metastatic sites [6]. Greenblatt et al. ventured beyond this convention, driven by the hypothesis that a unique stem cell might play a pivotal role in vertebral development [2]. Their findings, resulting from rigorous analysis of skeletal stem cells, have filled in some of the knowledge gaps, particularly in highlighting the differential gene activities of stem cells based on their bone origin [1].

As with all groundbreaking research, new answers often lead to further questions. For example, what sets vSSCs apart from other stem cells in terms of their properties, and how can we effectively utilize our newfound understanding of MFGE8’s role for therapeutic purposes? To address these questions, two key strategies emerge. First, inhibiting MFGE8 represents a crucial approach to mitigating the risks associated with spinal metastasis. Second, exploring the unique characteristics of vSSCs, particularly concerning spinal disorders, is imperative. As our journey continues, it becomes clear that the insights gained transcend mere academic exercises; they hold the potential to revolutionize treatment approaches and enhance patient outcomes.

## The Distinctiveness of Vertebral Skeletal Stem Cells

**Figure 1:**
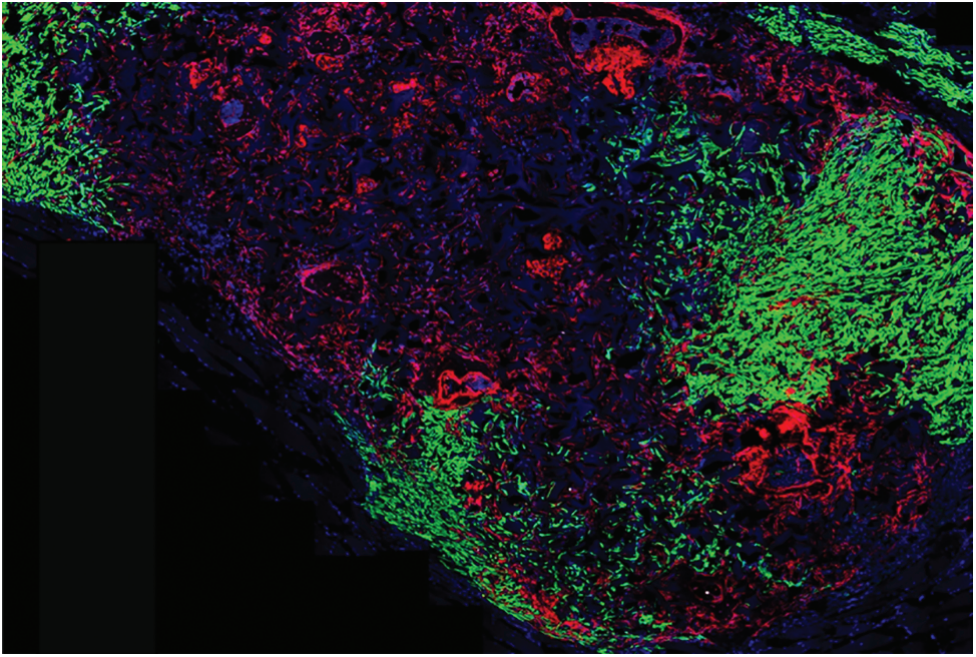
Spine stem cell recruitment of breast cancer cells. A new stem cell that forms the spine was transplanted into a model organism and allowed to form a miniature vertebral bone (red). Breast cancer tumor cells (green) invaded the bone, demonstrating that this new spine stem cell is responsible for recruiting breast cancer cells. Adapted from Jun Sun, Weill Cornell Medicine College, New York, USA [1].

Within the intricate landscape of the human body, specific cells (e.g., stem cells, etc.) stand out, not just for their rarity but for their unique roles that set them apart from the multitude. Among these are the vSSCs [1]. While stem cells, in general, have become synonymous with regeneration and potential, vSSCs claim their own niche, especially when it comes to the intricacies of vertebral bone health. Defined by a unique set of markers and characteristics, these cells exhibit properties that are both intriguing and significant. Beyond their defining characteristics, vSSCs play a specialized role in the physiology of vertebral bones, providing a framework for understanding their unique contributions and potential implications for bone health and disease (Fig. 1).

## Bone Metastasis—A Growing Concern

The skeletal system, with its intricate structure, serves not only as a vital support framework but also as an unwitting host to invasive cancer cells. One of the most formidable challenges in cancer progression is bone metastasis, where cancer cells migrate from their primary site to establish colonies within the bone. While this concern applies to the entire skeletal framework, the vertebral column is particularly vulnerable. Spine metastases account for a significant portion, approximately 70%, of all osseous metastases [7]. According to the Memorial Sloan-Kettering Cancer Center, as many as 40% of cancer patients may experience spine metastases [8]. Furthermore, for specific cancer types like breast and prostate cancer, spinal metastasis can be even more prevalent, with rates ranging from 70% to 90% (https://www.ncbi.nlm.nih.gov/books/NBK441950). These statistics underscore the alarming prevalence and impact of spinal metastasis, emphasizing the urgent need for intensified research and interventions. However, despite the increasing numbers, our understanding of the underlying mechanisms and factors responsible for this targeted invasion has remained unclear. Prior to recent research breakthroughs, significant gaps in comprehension persisted, posing challenges for clinicians and researchers alike.

## Connecting the Dots: vSSCs and Bone Metastasis

In the complex puzzle of bone metastasis, every discovery offers a piece that edges us closer to the complete picture. A cornerstone of this evolving image is the role of vSSCs. As researchers have delved deeper into the mysteries of bone metastasis, the influence and interplay of vSSCs have come to the forefront, shining a light on previously uncharted territories of metastatic pathways. The mechanisms through which these specialized cells might promote or facilitate metastasis are both fascinating and revealing, offering transformative insights into the metastatic cascade. Yet, what truly stands out about these recent revelations is not just their detail but their broader implications. They represent a pivotal shift in our understanding, reshaping the landscape of metastatic research and offering hope for novel interventions and therapeutic strategies.

## Therapeutic Horizons: A Beacon of Hope

Every groundbreaking discovery in medical research not only heralds novel therapeutic strategies but also ignites our collective hope for more effective therapeutic solutions. As we deepen our understanding of vSSCs and their crucial role in bone metastasis, we find ourselves at the dawn of a transformative era in therapeutic approaches. Envision a future where our comprehension of these cells enables the development of targeted genetic interventions that address metastasis at its core. Further, imagine leveraging advanced strategies to manipulate key components, like MFGE8, thereby potentially changing the course of bone metastatic diseases. Given these promising advancements, it’s conceivable to see a future where our methods for preventing bone metastasis and inhibiting its growth are not just enhanced but entirely revolutionized.

Recognizing the profound repercussions of skeletal metastases on patient health and longevity underscores the dire need for breakthroughs in bone metastasis prevention and growth inhibition. Historically, bisphosphonates have been our mainstay, recognized for their prowess in thwarting bone degradation [9]. These agents have been instrumental in managing skeletal complications across a spectrum of metastatic cancers, mainly through their action on osteoclast-mediated bone resorption [10].

Our insights into vSSCs offer a tantalizing prospect of synergizing these traditional treatments with new-age interventions. Among the most promising are strategies centering on vSSC-linked pathways, with a special emphasis on MFGE8. In this context, the investigation of a proto-oncogene tyrosine-protein kinase (Src) and its kinase inhibitor, Saracatinib (AZD0530), holds significant promise. Although Saracatinib has shown potential in curtailing osteoclast activity in patients with advanced cancers [11], it’s crucial to acknowledge its mixed results in clinical trials, especially its limited benefits in post-menopausal women with metastatic breast cancer regarding bone metastases [12].

As the realms of research expand, there is a palpable excitement for the emergence of innovative therapeutics. These anticipated candidates might not only amplify the potency of existing regimens but also chart previously unexplored mechanisms of action. This dynamic and progressive therapeutic milieu, laser-focused on the central challenge of bone metastasis, offers a beacon of hope for enhanced prognoses for individuals contending with metastatic bone disorders.

## Challenges and Considerations

In the momentum of scientific discoveries, it is easy to be swept away by the possibilities and promises they bring. However, with great innovation comes an array of challenges and considerations that must be meticulously addressed. Stem cell research, while a frontier of immense potential, also treads upon grounds laden with ethical dilemmas. The journey from laboratory benches to bedside therapies is not a straightforward path; it is riddled with technological and scientific hurdles that demand both creativity and caution. Translating the profound insights on vSSCs into tangible clinical applications requires not just scientific prowess but also a harmonious collaboration across disciplines. Bridging the gap between biology, ethics, technology, and clinical practice is imperative to harness the full potential of our discoveries.

## Conclusion and Future Directions

This pioneering study marks a significant leap in our understanding of bone metastasis, spotlighting the transformative role of vSSCs. Their implications extend beyond the mere deepening of our understanding; they present a roadmap for future therapeutic developments in the realm of orthopedic research. While the present findings are revolutionary, it is crucial to recognize that we stand at the threshold of this exciting journey. The myriad unanswered questions about vSSCs beckon intensified, dedicated research. There is potential for further exploration into how these stem cells interact with tumor environments, potential genetic modifications for therapeutic benefits, and the translation of these findings into tangible treatments for patients. Moreover, as the field progresses, collaborations across various disciplines, ranging from oncology to bioengineering, will be invaluable. Such interdisciplinary approaches could spark innovative strategies, enhancing the depth and breadth of our understanding and therapeutic applications. As we peer into the future, the horizon looks promising. This study has set the stage for an era where the potential of vSSCs might be fully realized, heralding a transformative impact on patient outcomes, and reshaping the contours of orthopedic research.

## Data Availability

The data and materials used in this study are available upon reasonable request from the corresponding author.

## Abbreviations

vSSCs: vertebral skeletal stem cells
MFGE8: milk fat globule epidermal growth factor 8
DEGs: differentially expressed genes
PI3K: phosphatidylinositol 3-kinase
MAPK: mitogen activated protein kinases
ERK: extracellular signal-regulated kinase
SRC: proto-oncogene, tyrosine-protein kinase
